# A60 INVESTIGATING THE ROLE OF NUCLEOTIDE-BINDING OLIGOMERIZATION DOMAIN (NOD) PROTEINS DURING BACTERIAL INFECTIONS IN INTESTINAL EPITHELIAL CELLS

**DOI:** 10.1093/jcag/gwad061.060

**Published:** 2024-02-14

**Authors:** M Nissan, S E Girardin

**Affiliations:** University of Toronto Temerty Faculty of Medicine, Toronto, ON, Canada; University of Toronto Temerty Faculty of Medicine, Toronto, ON, Canada

## Abstract

**Background:**

Inflammatory bowel disease (IBD) is a collection of conditions that result in the inflammation of the gastrointestinal tract causing diarrhea, abdominal pain, weight loss, nausea, and vomiting. With its prevalence a whopping 0.6% in Canada, there is a need to understand the targets of therapy for the disease. Crohn’s disease, which is one of the two main conditions implicated in IBD, is most often associated with polymorphisms in the NOD2 gene. NOD2 is integral to the innate immune system of mammals, and functions as a pattern recognition receptor within the cytosol of cells. It detects conserved bacterial peptidoglycans to elicit an anti-microbial response by activating the nuclear factor kappa B pathway. NOD proteins also tackle infections upon bacterial recognition by recruitment of autophagy proteins allowing for the degradation of invading bacteria. Given their roles in bacterial clearance, it is unsurprising that mice deficient for NOD1 or NOD2 show increased susceptibility to pathogens, which is also what is thought to occur with Crohn's disease patients.

**Aims:**

Although NOD1 and NOD2 have been well characterized in immune cells, little research has been done to interrogate their role during bacterial dysbiosis in intestinal epithelial cells. This research aims to elucidate the global signaling landscape that these proteins modulate in intestinal epithelial cells post-infection. We hypothesize that NOD1 and NOD2 play a critical role in host defense against intracellular bacterial pathogens in intestinal epithelial cells.

**Methods:**

Intestinal crypts were harvested from WT, Nlrc4^-/-^, and Nlrc4^-/-^ Ripk2^-/-^ C57BL/6 mice to generate primary ileal organoids. Organoids infected with *Shigella flexneri* were harvested for protein and RNA. Downstream western blot, qPCR, Immunohistochemistry, and CFU analyses were performed to ensure the efficacy of the infection.

**Results:**

Upon *Shigella flexneri* infection, CFU counts were only able to be recorded in a non-pyroptotic background (Nlrc4^-/-^). When examining the organoids by Immunohistochemistry, *Shigella* was well visualized in the Nlrc4^-/-^ enteroids and was absent in WT controls 4 hours post-infection. Additionally, an increase in pro-inflammatory and stress genes was seen only in Nlrc4^-/-^ organoids compared to WT controls. Finally, Nlrc4^-/-^ Ripk2^-/-^ organoids experience higher bacterial loads by CFU analysis compared to Nlrc4^-/-^Ripk2^+/+^ organoids.

**Conclusions:**

Our research has shown that NOD1 and NOD2 deficiencies in primary mouse epithelial cells have increased bacteria loads compared to WTs upon infections showcasing a novel epithelial-intrinsic role for NOD1 and NOD2. This new insight into an epithelium-intrinsic role may be a step towards understanding the mechanism behind NOD2-associated Crohn's disease pathogenesis.

**Funding Agencies:**

CIHRCrohn's and Colitis Canada

